# Open access and its potential impact on public health – A South African perspective

**DOI:** 10.3389/frma.2022.975109

**Published:** 2022-12-02

**Authors:** Adéle Strydom, Juanita Mellet, Jeanne Van Rensburg, Ignatius Viljoen, Anastasios Athanasiadis, Michael S. Pepper

**Affiliations:** SAMRC Extramural Unit for Stem Cell Research and Therapy, Department of Immunology, Faculty of Health Sciences, Institute for Cellular and Molecular Medicine, University of Pretoria, Pretoria, South Africa

**Keywords:** open science, open access, publication costs, privacy, POPIA, secondary use of data

## Abstract

Traditionally, access to research information has been restricted through journal subscriptions. This means that research entities and individuals who were unable to afford subscription costs did not have access to journal articles. There has however been a progressive shift toward electronic access to journal publications and subsequently growth in the number of journals available globally. In the context of electronic journals, both open access and restricted access options exist. While the latter option is comparable to traditional, subscription-based paper journals, open access journal publications follow an “open science” publishing model allowing scholarly communications and outputs to be publicly available online at no cost to the reader. However, for readers to enjoy open access, publication costs are shifted elsewhere, typically onto academic institutions and authors. SARS-CoV-2, and the resulting COVID-19 pandemic have highlighted the benefits of open science through accelerated research and unprecedented levels of collaboration and data sharing. South Africa is one of the leading open access countries on the African continent. This paper focuses on open access in the South African higher education research context with an emphasis on our Institution and our own experiences. It also addresses the financial implications of open access and provides possible solutions for reducing the cost of publication for researchers and their institutions. Privacy in open access and the role of the Protection of Personal Information Act (POPIA) in medical research and secondary use of data in South Africa will also be discussed.

## Introduction

Traditional subscription-based publishing models where individuals or institutions pay subscription fees in order to gain access to scientific material have been the modus operandi for many years. More recently, “Open Science,” a global movement that aims to make the conduct and dissemination of research methods and results accessible to all, has been gaining traction. This is done in order to promote transparency and collaboration to the benefit of the global community (Besançon et al., 2021), and is applied through Open Science practices that include open access, open source, open data, open methodology, and open peer-review. Open access strives to remove the financial and legal restrictions that can prevent individuals from accessing research publications and outputs (Prlic and Procter, 2012; Tennant et al., 2016; Muñoz-Tamayo et al., 2022; UNESCO, 2022a). Open access can also include early distribution of manuscripts in the form of preprints, in other words, draft articles that have not yet been peer-reviewed or published in scientific journals. Open source, open data and open methodology ensure that all research data and the various tools used to acquire and analyze the data are shared in an unrestricted manner, thereby promoting and facilitating the rapid replication of studies, increasing re-use of data, and assisting in the peer-review process. Open source, open data and open methodology should provide data publicly, and without cost and access restrictions (York, 2022). Open peer-review allows for the public sharing of peer-review reports and author responses in a transparent manner. This practice maintains a high quality of peer-review and reduces the risk of hidden conflicts of interest (Szekely et al., 2014).

The increase in online journals in the 1990s initiated the open science movement with the purpose of supporting transparency and collaboration in research and scientific communication (Huston et al., 2019). In public health, open science provided benefits such as opportunities for scientific collaboration and partnerships, increased research and analytical capacity, early detection of health and environmental threats, monitoring of real-time response, informed policy decisions, more capacity for public participation, transparency and better accountability. The COVID-19 pandemic highlighted the benefits of open science but also exposed the challenges related to the accuracy and validity of scientific information (Besançon et al., 2021). During the pandemic, researchers relied less on traditional systems of publishing and embraced open access platforms and preprint repositories to disseminate their COVID-19-related research results as quickly as possible. A preprint is a version of a scientific manuscript posted on a public server prior to formal peer review. Even though preprints may contain errors and potentially increase the risk of disseminating misinformation, they provide an open and transparent publication mechanism, thereby accelerating communication between scientists.

Although COVID-19 exposed the need for open science, open access to scientific knowledge is still a dilemma for many scientists, especially in resource-constrained countries. Scientific studies can consequently become locked behind subscription paywalls thereby blocking those lacking appropriate journal subscriptions or financial resources from obtaining access to research material (International Science Council, 2022). Except for diamond publishing where authors do not pay for open access publishing, the typical gold open access model affords access to publications but transfers the responsibility of payment from individuals to academics/authors and their institutions. While it is recognized that open access to peer-reviewed publications is critical for scientific advancement and affords readers unrestricted access to information, payment is still required to cover costs relating to editing, typesetting, printing, binding, marketing, distribution and archiving (The Conversation Africa, 2022b).

Despite inadequate funding and limited research capacity, African scientists have made valuable but limited contributions to COVID-19 research. Two studies concluded that only 3% of all COVID-19-related articles (not including preprints) were authored by African scientists and just 4.3% contained information specific to Africa and/or African countries (Kana et al., 2021; Naidoo et al., 2021). South Africa, Egypt and Nigeria collectively provided 65% of the COVID-19 articles produced by Africans. According to Naidoo et al. (2021), one in five African COVID-19 papers had no African authors, and approximately 66% of authors on papers with research originating from African populations were non-African nationals. Both these studies highlight the need to boost the research production by African scientists and the need to support the publication of their research findings.

South Africa is considered a pioneer in Africa regarding open access policies and the structures that enforce them (UNESCO, 2017). As of February 2022, South Africa had indexed more than 100 South African-based open access journals in the DOAJ (2022) (Directory of Open Access Journals). Open data and the sharing of health data for research should nevertheless be subjected to legal and ethical procedures (Staunton et al., 2021), especially for secondary use of such data. This has changed notably in South Africa since the implementation of the Protection of Personal Information Act (POPIA) No. 4 of 2013.

This paper will discuss the opportunities and challenges associated with open access to research in the South African higher education context. It will also address the financial implications of open access to academic institutions, amongst other role players, and provide possible solutions to reduce publication costs for researchers and their associated institutions. Since South Africa is classified as an upper middle-income country (UMIC; The World Bank, 2022) and considering the limited research resources available, this article will also address the publishing policies and journal selection processes that need to be considered when publishing open access. Privacy in open access and the role of the POPIA in medical research and secondary use of data, will also be discussed.

## Open access – Opportunities and challenges

There are multiple journal ranking systems that serve as proxies for the quality of a given journal. The most utilized is the impact factor (IF) which is the ratio of citations to the number of citable publications published by a given journal. It is calculated based on a two-year period where the number of annual citations is divided by the number of publications in the previous 2 years (Sharma et al., 2014).

Each article undergoes a rigorous peer-review process. Peer-review is a process in which experts in the field under consideration serve as a quality control checkpoint where scientific processes and claims are verified, rendering approved articles scientifically reliable and valid. The higher the quality of the journal, the more arduous the reviewing process (making it difficult to publish in highly revered journals). Credibility is often reflected by the number of citations a publication has received, which in turn, increases the journal's IF.

Dissemination of research information can be accelerated through preprints and expedited peer-review and publishing processes, as was observed during the COVID-19 pandemic.

Journals indexed in major databases such as Medline, Scopus and PubMed are considered to be of acceptable scientific quality. A journal's publishing history and scope are likewise considered important and are reflected in the journal's impact. These measures affect the relative importance of journals in a field and also reflect the journal's reach. When deviating from these well-established publication practices, publications may end up in predatory journals. “Predatory” journals and publishers make false claims and provide misleading information to manipulate authors into publishing therein. Several predatory journals have author-pays practices and should be avoided, since publishing in such journals may harm the reputation of the authors and their affiliated institutions, and provides a highly questionable way around the all-important peer-review process.

This section serves to explore themes associated with open access publications, preprints and predatory journals.

### Open access publications

Medical journals such as the New England Journal of Medicine, the British Medical Journal and the Journal of the American Medical Association are published in both paper and electronic formats. These journals rely on association membership fees and journal subscriptions to cover publication costs. With the growth of digital media, publication cost and revenue models have changed, and new funding models have had to be developed. Publishing open access allows readers to have immediate access without the need for subscriptions through institutions. Various types of open access models exist, and includes gold, green, bronze, hybrid, and diamond. The majority of open access journals, with the exception of the latter, incur a cost to the authors and/or funders for peer-review and publication, referred to as article processing charges (APCs). Gold open access is when authors and funders pay for published articles to allow immediate access without any restrictions. With green open access, a self-archived version of the manuscript is made available through an open access repository or website (Piwowar et al., 2018). In the case of an embargo period (~6–12 months), readers are required to pay a fee to access these articles during that period. In 2018, the majority of open access articles were published as “bronze”. Articles published under this open access model are free to read on publisher websites but do not have a formal license for reuse (Piwowar et al., 2018). Hybrid journals charge APCs in addition to subscription costs that allow readers to access the full contents of the journal (Piwowar et al., 2018). At the extremes of the spectrum, we have pure subscription journals and pure “pay for publication” journals. In-between, some subscription journals offer free “green open access” and paid “gold open access” options. A wide range of hybrid journals offer subscriptions, site licenses and pay-for-use to readers, with different paid open access options to authors.

In the South African context, publishing research in a journal accredited by the Department of Higher Education and Training (DHET) has benefits for both the researcher responsible for the article as well as the institution they belong to. Increased access and readership will likely result in improved citation that might lead to wider visibility for authors and their institutions (Shuai et al., 2012). The number of articles that researchers publish in reputable journals is taken into consideration by academic and private institutions when performance-related decisions are made. Additionally, in the greater scheme of science and information sharing, it is through publication that information circulates through to others in the field, expanding the pool of knowledge and ultimately advancing scientific progress. Additional benefit is derived by South African academic institutions when publishing research in accredited journals, as these publications receive a subsidy from the South African government through the DHET (Woodiwiss, 2012). South Africa's experience with open access publishing is not new and the issues about high publishing costs have been discussed by other authors (Czerniewicz and Goodier, 2014; Hartman and Wu, 2018; Bawa, 2020).

Currently, journal articles have a system of reviewer recruitment which usually does not benefit the reviewer. However, depending on the publishing authority, reviewers may benefit through the wavering of APCs for articles that they wish to publish in the future. In some instances, the reviewers' contributions may be uploaded onto a commercial website named Publons (2022). Publons is a free platform that ensures recognition for peer-review and editorial contributions. Publons generates a review record that can be used in CVs, job and funding applications, and performance evaluations.

Lastly, publishing institutions benefit through the sale of published articles, and monthly/yearly subscriptions from academic institutions from which the author and/or reviewer benefit as a consequence of exposure rather than financial gain.

### Preprints

More than 80,000 COVID-19 and SARS-CoV-2-related preprints and peer-reviewed articles have been published since the emergence of this virus in December 2019 (Besançon et al., 2021). In 2020, preprints accounted for 17–30% of all COVID-19 research papers (Else, 2020). In South Africa, the first COVID-19 case was reported in March 2020 and the Network for Genomic Surveillance in South Africa (NGS-SA) was created to investigate the dynamics of the SARS-CoV-2 epidemic (Giandhari et al., 2021). Consequently, preprints on South African SARS-CoV-2 variants and the immune response have been posted since 2020 (Callaway, 2021; Giandhari et al., 2021). Overall, preprints have accelerated the pace of research, and public-health policies have been directly informed by them. The use of preprints may thus be beneficial, but their unfettered use also raises concerns (Horby, 2022). Although preprints enable quicker data sharing during a crisis and allow scientists to improve their work with informal feedback, it also opens the door to use of predatory journals (Watson, 2022) and the use of social media to disseminate preprint findings (Koerber, 2021). Unfortunately, rapid preprints potentially increase the risk of fraudulent, deceptive or poor quality research (Horby, 2022). This may result in premature and misguided claims and increased confusion, especially when the distinction between preprints and standard peer-reviewed articles is poorly communicated or misunderstood by the media and the public. Publications in predatory journals that are not peer-reviewed may endanger public health by publishing inaccurate and unvalidated findings (Watson, 2022). This may contribute to unreliable meta-analyses, or flawed research data and findings.

Preprint publications contributed important knowledge on COVID-19, despite possibly having methodological weaknesses that could limit the interpretation of the results and provide misleading or false claims that could greatly impact public health (Besançon et al., 2021). Despite the fact that some studies were retracted, their claims still contributed to the body of knowledge. As a case in point, when the retraction rate of COVID-19-related articles was compared to publications in related research fields, the authors concluded that the rate was approximately four retractions for every 10,000 papers. This may have been the result of researchers rushing to submit manuscripts for publication and the expedited peer-review and publication process of COVID-19 articles by some journals (Yeo-Teh and Tang, 2021). This poses a direct threat to public health and leads to wastage of scientific resources and public confusion (Besançon et al., 2021). While academic communities rapidly disputed false claims, public perceptions are influenced by the dissemination of preprint information in mainstream media articles (Brierley, 2021; Fraser et al., 2021). The World Health Organization (WHO) has been raising awareness about an “infodemic” as social media has amplified and exacerbated misinformation and uncertainty (Vraga et al., 2020). Promoting news and science literacy allows people to determine whether information about COVID-19, or any other disease, is accurate, and empowers them to take active control of their social media feeds and protect themselves and others.

The quality of research becomes questionable when peer review is absent. This has the potential to drive negative perceptions, particularly within clinical research, and may therefore impact the sector as a whole, and not just at an individual level (Kwon, 2020). Consequently, preprint servers, such as BioRxiv (2022) and MedRxiv (2022), now have enhanced screening procedures in place. Both bioRxiv and medRxiv screen articles in a two-tiered approach which firstly requires that in-house staff examine the manuscript before seeking expert opinion concerning scientific merit and validity. This is done in order to ensure that scientifically sound, original research is being placed in the public domain. Health professionals and principal investigators are primarily used to review submitted preprints in medRxiv and bioRxiv. While the former (in-house) screening requires more time to complete, the latter is typically finished within 2 days. Since papers in medRxiv may be more relevant to health, they are scrutinized more closely and therefore take longer to evaluate. Rather than determining research quality, vetting is primarily used to identify potentially harmful articles, including those that do not provide evidence-based conclusions and/or make statements viewed as contradictory without just cause (Kwon, 2020). Recently though, this vetting process has been extended to exclude computational modeling papers considered to be “speculative” in nature.

Therefore, to ensure good quality research through open science initiatives, this process must go hand in hand with, for example, full data sharing and the publication of study protocols approved by International Regulatory Boards or Research Ethics Committees prior to the initiation of clinical trials (Watson, 2022). Similarly, use in policy decisions and modeling must be undertaken with caution and be transparent, while preprint servers may need to put additional screening measures and procedures into place to block the distribution of poor-quality manuscripts. Disclaimers relating to the preprint status of articles could also be used to combat publishing of preprints by predatory journals.

### Predatory journals

In March 2022, the InterAcademy Partnership (IAP) published a report entitled “Combatting Predatory Academic Journals and Conferences” (Inter Academy Partnership, 2022). One of the main aims of the report was to find ways to prevent and reduce the number of predatory journals and conferences. According to the report, there are over 15 500 predatory journals around the world with widespread predatory practices. Predatory practices such as phishing, being misleading by using false identities or re-publishing papers already in legitimate journals without permission will continue to be fueled by the digitization of academic publishing, gold and author-pays open access publishing models, and research evaluation criteria that emphasize quantity over quality. Authors have succumbed to predatory journals who seek papers *via* email and social media, promising to publish open access articles rapidly and with minimum review, frequently for a charge (Vervoort et al., 2020). The “publish-or-perish” mindset in academia, combined with obstacles to publish encountered by researchers from low to middle income countries (LMICs) has resulted in a rapid increase in the number of predatory journals which are easy to publish in (Vervoort et al., 2020). This affects the visibility of credible scientific research, stifles scientific progress, and jeopardizes the reputations of authors and the institutions they represent.

According to a previous study, South African academics published 728 articles in only five predatory journals over the three and a half years covered by the study (De Jager et al., 2017). This may have been due to local researchers collectively legitimizing these predatory journals. In this study, the level of predatory publications was found to have financial implications. Using an estimated subsidy of ZAR100 000 per article as an example, it was estimated that a total of ZAR70 million might possibly have been paid for publications in journals that did not meet strict academic research quality criteria. A combination of the South African subsidy system, the involvement of South African academics on editorial panels and reviewer lists, promotional material, and a substantial amount of South African written articles in such publications, had to some extent legitimized these journals in the South African system.

Using open access policies, funders can encourage funded researchers to publish in credible journals that adhere to established open access principles (Shamseer et al., 2021). Open access is not understood by most researchers beyond making research free to read. Journals with deceptive or nefarious publishing operations might have gained from or taken advantage of the inexperience of some authors. Predatory journals do not always include licensing information for articles or provide information on reusing published research. Scientists who publish in predatory journals are likely to be violating their funders' open access policies (Shamseer et al., 2021).

## APCs and publisher profits

### Article processing charges

In contrast to subscription-based publication models whereby publication costs are absorbed by the reader or their institution, the cost of open access models to academics is substantial. As an example, Nature and Lancet respectively charge academics €9 500 and US$5 000 per open access paper (The Conversation Africa, 2022b). Both Nature and Lancet call this APCs while other journals refer to a “publication fee”. In Africa, the publication costs to researchers are hugely burdensome as a consequence of currency exchange rates. To demonstrate this practically, at an approximated exchange rate of ZAR17,00 to €1 and ZAR16,00 to US$1, the APCs in South Africa would be the equivalent of paying ZAR161 500 and ZAR80 000, or 16.2 and 8.0% of a one million Rand budget, respectively. This is equivalent to a €9 500 cost on a €60 000 budget, or US$5 000 cost to a budget of US$62 500. Several LMICs have weaker currencies when compared to South Africa. This further demonstrates the potential cost implications for researchers in LMICs and the financial pressure that many LMIC universities and associated researchers face with regard to APCs.

The authors of this paper are all members of the Institute for Cellular and Molecular Medicine (ICMM) of the Department of Immunology in the Faculty of Health Science at the University of Pretoria, South Africa. The ICMM is a transdisciplinary, translational, highly collaborative entity that aims to understand and manage specific contributors to the disease burden in South Africa and Africa. Active research projects cover a wide range of disciplines and entities, with a particular focus on molecular, cell and computational biology, and the ethical, legal, and social implications of research in cell and gene therapy. The ICMM comprises senior researchers, post-doctoral scientists, and postgraduate students of medical, scientific, ethical, data sciences and legal disciplines. Research papers are prepared and submitted for publication to a wide spectrum of academic journals.

African research groups are uniquely positioned and able to more accurately perform, collaborate with, sustain and describe research endeavors in Africa (Kana et al., 2021). However, as alluded to previously, this comes with notable costs to researchers. To demonstrate this practically, APCs paid during the course of 2021 for 10 publications associated with authors representing the ICMM totaled nearly ZAR330 000. This was distributed across APCs that were charged in Swiss Francs, Euros, and US Dollars. As shown in Table 1, the exchange rates increased the relative Rand (ZAR) cost of the APCs anywhere between 8.6 and 17.7 times that of the foreign currency equivalent. The average APC per published article was ZAR32 803,94, with the total costs representing nearly 33% of a ZAR 1 million budget. While not reflected in Table 1, institutional contributions totaling R86 075,00 were received during the 2021 period for six of the 10 articles with contributions still pending for the remaining four publications. While institutional contributions may cover up to 50% of the APCs, they are not guaranteed and can also take several months to reflect in research accounts.

**Table 1:** A summary of the APCs paid for 10 published articles during 2021.

	**CHF (*N*= 4)**	**EUR (*N*= 1)**	**USD (*N*= 5)**
Min cost per article	CHF 1 440.00	N/A	USD 1 867.50
Max cost per article	CHF 1 800.00	N/A	USD 2 950.00
Mean cost	CHF 1 710.00	N/A	USD 2 243.50
Total cost	CHF 6 840.00	EUR 3 582.25	USD 11 217.50
Min exchange rate (ZAR:1 FC)	ZAR 8.59	ZAR 16.12	ZAR 14.51
Max exchange rate (ZAR:1 FC)	ZAR 17.74	N/A	ZAR 15.54
Mean exchange rate (ZAR:1 FC)	ZAR 14.79	N/A	ZAR 15.10
Min cost in ZAR	ZAR 15 469.90	N/A	ZAR 28 279.16
Max cost in ZAR	ZAR 31 923.49	N/A	ZAR 45 853.03
Mean cost in ZAR	ZAR 25 132.55	N/A	ZAR 33 955.48
Total cost in ZAR	ZAR 100 530.18	ZAR 57 731.88	ZAR 169 777.38

ZAR, South African Rand; FC, foreign currency; CHF, Swiss Franc; EUR, Euro; USD, US Dollar.

While reimbursements and financial “rewards” for publications are provided at some academic institutions, the financial resources to do so must come from somewhere. The University of Pretoria, as with most other academic institutions in South Africa, receives a subsidy per publication in an accredited journal from the South African government on an annual basis through the DHET. This subsidy is allocated to the Institution, Faculties, Schools, Departments and ultimately to academics according to various payment structures determined by the institution itself. Funding to academics who raise the funding, do the research and publish their manuscripts is on an *ad hoc* basis and may only be sufficient to cover the cost of one or two manuscripts. These payment structures vary across academic institutions and do not appear to be consistently observed. They are also restricted according to the availability of funds. Internally, the University of Pretoria open access fund provides partial support for APCs for articles published in accredited open access journals when no alternative funding is available. This support is based on a set of criteria provided in guidelines for applications to the fund. These criteria and the proportion of the APCs to be refunded per article may be reviewed and revised. The fund is supported by a reserve set aside from the annual resource allocation provided to the Department of Library Services. The fund does not support APCs for hybrid (open choice) journals and excludes monographs, book chapters and publications. Since support is dependent on the availability of funds, payment is not guaranteed. As such, the university urges researchers to request a waiver of APCs from target journals and to request APC support from the library services before submitting articles to journals.

While we have some appreciation of APCs relative to South African institutions, the true cost of open access for researchers based in LMICs is largely unknown (The Conversation Africa, 2022b). An urgent discussion is therefore needed on the financial implications of open access publishing for academic and research institutions in Africa. The rising cost of journal subscriptions is leading to an increasing number of questions concerning the academic publishing establishment (Van Noorden, 2013), and while some journals consider waiving the costs for 47 historically disadvantaged academic institutions in LMICs, research institutions from the remaining 58 nations are expected to cover all or reduced publication costs (The Conversation Africa, 2022b). This potentially creates a conundrum for researchers when applying for research grants to cover the costs of open access publications. Since grant funding is limited, researchers must carefully consider what they wish to publish and how they wish to publish prior to commencing the research; there is usually little room to adjust this plan once grant funding is approved. Using the costs incurred by the ICMM for 10 open access articles published in 2021 as an example, it is clearly not feasible to request support for open access publications through grant applications valued at less than R100 000 when the average cost of a single article in a medical sciences discipline would likely cost 30–50% of the total value of such a grant.

### Publisher profits

While the philosophy behind open access requires that authors retain their copyright, in practice researchers and their institutions are required to assign copyright to the publisher in addition to paying APCs to publishers who generate profits through this process. Peer-review is typically also done without compensation. This has created an “asymmetric business model” (International Science Council, 2022). What contributes to the high cost of open access publishing? Commercial, profit-driven publishing houses sell journal subscriptions and site licenses or charge pay-per-use fees. They also apply embargoes that may range from 6 months to potentially indefinite periods of time where they own the copyright. All of these journals claim to be “open access”. While “green open access” options are seldom provided, journals require payment of APCs to give readers “gold open access”. This has made academic publishing a lucrative business. As a case in point, it was reported that the revenue generated by the scientific publishing industry in 2011 totaled US$9.4 billion. This included the roughly US$5 000 generated per article of the nearly 1.8 million English-language articles published at that time (Van Noorden, 2013). The total revenue generated by the academic publishing industry increased to more than US$19 billion in 2017 (Hagve, 2020).

Over 50% of the publication industry is dominated by five publishers. These include Elsevier, Black and Wiley, Taylor and Francis, Springer Nature, and SAGE. Approximately 16% of the market is held by Elsevier, which publishes more than 3 000 academic journals. Unlike companies such as Microsoft, Google, and Coca Cola, Elsevier boasts a profit margin approaching 40% and the curve indicates an upward trend (Hagve, 2020). Between 1991 and 2013, profit margins for all of Reed Elsevier's enterprises ranged between an estimated 14 and 27% (Larivière et al., 2015), which aligns with the average estimated 20–30% profit margins currently noted for other publishers. However, when focusing on their “Scientific, Technical and Medical division,” profit margins across the 1991–2013 period reportedly ranged between an estimated 30 and 42% while operating profits at the same time-period ranged between approximately 9 (1991) and 43% (2013). Operating profits were noted to have a strong upward trend between 1991 and 2013 (Larivière et al., 2015).

To place this into context from a South African perspective, at an approximate exchange rate of ZAR20.00 to £1.00, Elsevier's recently reported revenues of £2.64 billion and net profit of £1.922 billion (72.8%) equates to a staggering revenue of ZAR52.8 billion, and net profit of ZAR38.44 billion. This represents roughly 95% of the ZAR40.4 billion and 15% of the ZAR259 billion budget respectively allocated toward provincial hospital services and the entire public health sector for the 2022 financial period by the South African Treasury [National Treasury (RSA), 2022]. Representing the South African public hospital industry, the provincial hospital services sector provides hospital care to approximately 80% of the South African population (South African Government, 2022). Given that the South African population comprises just over 60 million people (Statistics South Africa, 2022), this implies that the provincial hospital services sector must serve just over 48 million people with a budget that is a mere 5% more than recently reported net profits reported by Elsevier.

As a consequence of high profit margins, there has been a proliferation of publishing houses and journals and the capacity for academic journals to turn the situation of production costs on its head. For example, a traditional newspaper, whose profit tends to be 10–15%, incurs expenses for wages for its journalists, editors, and graphic artists, as well as expenses for research, printing, and distribution, all paid through sales and advertising. In the case of academic journals, production cost are paid for by research funds, researcher salaries, and the costs involved in undertaking research. However, academic editors receive symbolic pay, as quality control and fact-checking are done through peer-review, which is voluntary. Most of the access is digital, and therefore the only real cost incurred by the publisher is for graphic design of the article (Hagve, 2020).

For publishing houses, open access has provided a new way to generate a profit. However, it comes at a high cost to authors, with the price of publication often ranging from US$1 500–US$3 000 in a fully open access journal, and up to US$6 000 in traditional subscription journals. Although open access fees are transparent, revenue may also be generated through other means (Van Noorden, 2013). These include revenue generated through membership or subscription fees and subsidies that may be received, notably by smaller publishers. Subscription-based journals may additionally derive their revenue from cross-subsidies, by offering advertising opportunities, and charging fees for reprinting of articles. One reason for the lower costs of purely open access publishers is that they are providing a digital product from a business model that is less established than the traditional, subscription- and paper-based journals. Unlike their more established counterparts, this creates flexibility regarding presentation of their articles and subsequent reduction in production costs.

Ultimately, the success of publishing houses is dependent on how well their products sell. This is in turn dependent on the quality of their products. The quality of academic journals is measured through an IF, with a high IF being important for financial success. As indicated previously, the journal IF is based on the number of citations a journal's articles receives in a given period of time, with frequent citations increasing the journal's perceived importance and value (Hagve, 2020). The ‘exclusivity' of journals, as measured by their rejection rate, has also provided grounds for increased publication fees. The rejection process may prompt authors to consider alternative journals. Since journals perceived as being of greater value will naturally receive a greater number of submissions, publishers have argued that this is essential for researchers whose task is to sort through millions of published articles each year to determine which are worth reading.

### University libraries and their relation to APCs

The South African National Library and Information Consortium (SANLiC) is a non-profit organization that facilitates the process involved in obtaining licensing agreements for electronically accessed information. Members of SANLiC notably include public higher education and research institutions (SANLiC, 2021). Furthermore, SANLiC is committed to promoting open access for South African research outputs by increasing access to scholarly information, reducing the cost of library subscriptions, and looking for alternative forums for the distribution of South African scholarships. SANLiC has been successful in lowering the cost of subscription access for member libraries resulting in the expansion of their collections. In 2019, an overall 87.4% cost avoidance on subscription list prices was negotiated. Unfortunately, according to the organization, this is not enough to address the unsustainability of the entire pay-to-read model. The main annual expenditure on scholarly literature by South African higher education and research institutions is allocated to pay-to-read subscriptions and approximately 80% of these subscriptions are based on deals negotiated by SANLiC. In 2020, SANLiC spent US$27 130 138 (82% of their journal expenditure) on Big Five journal packages (Elsevier's Science Direct, Wiley, Springer Nature, Taylor and Francis, and SAGE). Only 52% of South African research output, i.e., research with South African corresponding authors, is published in journals covered by these packages. SANLiC did a data analysis in 2020 on research and review articles published between 2014 and 2019, and of the 62 549 publications assessed, approximately 33% were open access, while the remainder were behind paywalls. Recently, SANLiC signed a number of Read and Publish Agreements on behalf of South African higher education institutions. The list of current negotiated agreements is now available, thus facilitating publication in gold open access journals.

The total amount that South Africa spends on journal subscriptions is unknown. This is largely due to the fact that university libraries, research departments, and research institutions each have their own budgets for this. Additionally, there is a lack of transparency regarding publisher fees owing to nondisclosure agreements signed by research institutions (Mail and Guardian, 2022; The Conversation Africa, 2022a). It is important for academics to publish their work, not only to advance their careers, but also to increase research citations and visibility. This is a long-running issue between researchers and publishers, as journals make their profit from research while restricting access to it (Mail and Guardian, 2022). According to Elsevier, the embargo period in green open access journals is justifiable on the basis that it is (1) not uncommon practice to have embargo periods of 12–14 months for publications, and (2) that the publisher requires revenue from subscriptions to compensate for the publishing costs (Mail and Guardian, 2022). While implementation of embargo periods is not new, Elsevier's updated regulations regarding embargoes resulted in a petition being launched against it. Signatories of this petition not only include SANLiC and SANLiC affiliates, but also non-SANLiC members (Mail and Guardian, 2022). The signing of this petition was largely driven by the principles governed by open science, notably in the form of open access.

According to a 2015 White Paper published by the Max Planck Digital Library, it has been suggested that scientific subscription journals should alter their business models to adopt open access business models instead (The Conversation Africa, 2022b). It has also been suggested that such changes should be reflected in how countries challenge the publisher costs through amendments to their legal and financial structures (Schimmer et al., 2015). This may not be easy to implement in countries that like South Africa have little published data on fees charged by publishers, how much is actually spent on various publishing fees, or what discounts, waivers and/or rebates are granted by publishers. Nevertheless, through a 2018 survey to which 15 of the 26 South African public university libraries provided a response, it was found that more than ZAR1 billion (US$68 020 593) was paid toward fees for e-resources, book budgets, and copyright licenses (The Conversation Africa, 2022a). As a result of the increasingly unfavorable foreign exchange rate, it has been speculated that this amount may increase by about 5% per annum. Additionally, 14 of the 15 institutions pay roughly ZAR31 million (US$2 106 307.37) for copyright licenses on prescribed works. While limited, the noted expenditure for research and teaching purposes should be a major concern, especially when considering that an estimated 80% of literature purchased for use in academic libraries is produced by international publishers. Furthermore, a great portion of locally produced research is made visible through the publication of work using international publishers (The Conversation Africa, 2022b). More financial data is however required before the combined efforts aimed at impacting these costs can be experienced by researchers and associated institutions in LMICs.

In order to fully benefit from the principles that govern open access, university libraries in Africa have actively promoted the open access movement in a variety of ways. This has not only been seen within the academic research sector through the establishment and maintenance of institutional repositories (IRs) but has also included the cataloging of journals that facilitate and promote open access publications by University library services (Research Gate, 2022). Assuming that they are properly maintained, IRs therefore have the capacity to increase the visibility of research activities and outputs achieved by academic institutions. While it is noted that this information can change rapidly owing to daily revision of IR data, according to OpenDOAR, eight African countries currently have IRs. These include South Africa, Kenya, Nigeria, Algeria, Tanzania, Zimbabwe, Sudan and Ghana. IRs total 48, 44, 31, 20, 14, 11, 12, and 6 per country, respectively (OpenDOAR, 2022). The University of Pretoria hosts both a Research Data Repository (Figshare, 2022) and an Institutional Repository (UP Space, 2022). Both are operated by the Department of Library Services.

The standard means of accessing journals at present is through academic library subscriptions and private purchases. Due to paywalls that continue to frustrate access to journals and articles, multiple alternative options have been developed to provide free access. Sci-Hub is a controversial website that has emerged as a consequence. Sci-Hub (2022) provides mass public access to research papers located behind paywalls thus enabling the free sharing of information. However, this platform is illegal as it allows copyright infringement. A non-controversial version of Sci-Hub, named Unpaywall (2022), is a legal, commonly utilized browser extension of Google Chrome and Firefox that provides access to a repository of freely available scientific articles. When encountering an academic article online, a pop-up will appear providing the option to download the article for free if the article is available on Unpaywall.

### Institution-specific policies on open access and publishing

In October 2021, the University of Pretoria approved a Policy on Open Access to Research Papers and Creative Outputs Authored by University of Pretoria Researchers. While the policy itself is not publicly available, the purpose of the policy can be summarized as follows: (1) “to support several Open Access initiatives, including the Berlin Declaration”; (2) “to ensure that research conducted at the University is conducted to the highest possible standard, is made freely available to increase local and global visibility, and facilitates greater research impact and benefit to all stakeholders”; (3) “to honor ethical research and business standards, contractual obligations, legal restrictions, archiving requirements of research funders, and publishers' copyright regulations”; (4) “to support global initiatives to influence the current copyright practices of publishers and authors thereby expanding the rights of its authors and researchers”; and (5) “to provide directives for the archiving and dissemination of academic journal articles, conference papers and creative outputs authored or co-authored by University of Pretoria researchers which have been or will be published”.

The aim of this policy is to ensure that all published University of Pretoria research and creative outputs are available for use within the University and are freely accessible to any other student, researcher, or member of the public with a non-commercial requirement for access to the information. The policy applies to all postgraduate students, research staff, employees, visiting researchers, and postdoctoral fellows engaged in publishing and/or disseminating research outputs under the auspices of the University, even when they co-author with researchers from other institutions. Other sections in the policy include a policy statement, definitions, associated documents, roles and responsibilities of authors, Deans of Faculties, and the Department of Library Services, and describe where it is not applicable as well as the consequences of non-compliance. The policy is reviewed every 3 years.

In July 2014, the University approved their Policy on Open Access Publishing Processing Charges (UP Policies, 2022). The purpose of this policy is to facilitate open access publishing of research by students and staff at the University. It provides the principles for support of open access publishing by researchers and the criteria for funding of APCs through an Open Access Fund. Support is provided for articles to be published in peer-reviewed, international open access journals. A list of eligible open access journals, together with their IFs, is provided *via* restricted access by the Department of Library Services. This policy is also reviewed every 3 years. The current policy was reviewed in 2018 and remains unchanged.

In keeping with the objectives of the indicated policies, the Department of Library Services, as part of SANLiC signed transformative (read-and-publish) agreements in March 2022 with the following publishers: Wiley, Emerald, and Association for Computing Machinery (ACM; UP news, 2022). The main benefit is that publications in journals from these publishers are not subjected to APCs. The Department of Library Services has further expanded its services to support researchers to publish open access articles for free in hybrid journals from these publishers and has compiled a list of accredited journals which are part of these agreements. The list will constantly be updated as negotiations with publishers on transformative agreements are ongoing. In the meantime, authors can start submitting their manuscripts to Wiley and Emerald. Subscription to the Wiley hybrid open access journals provides read access and enables eligible corresponding University of Pretoria authors to publish articles at no extra charge. However, publishing in fully open access journals with Wiley is not free and may require authors to pay publishing fees. Regarding Emerald hybrid journals, research can be published through prepaid open access publishing vouchers if journals are eligible. All ACM open access journals publish articles for free.

## Privacy, POPIA, and research ethics

### Privacy and the impact of POPIA on open data sharing

Governments have recognized the value of open science and open access, particularly as they pertain to biological samples and their associated data. South Africa is no exception, with the South African Department of Science and Innovation recognizing its pertinence to genomic research and the Fourth Industrial Revolution (Staunton et al., 2019). For example, the sharing of genomic data has several benefits. These include ensuring the optimal use of resources such as facilitating studies that require larger sample sizes to ensure that they are statistically more powered, thereby facilitating reproducible research, creating new research opportunities from pre-existing data sets, and promoting research innovation. Notably, open data initiatives have often been considered important to ensure that a replication crisis does not occur, even if sharing raw data may be difficult due to compliance with data protection regulations in medical research. Despite this difficulty, they are often seen as an essential component of the peer-review system. Except where sharing of data is prohibited for privacy reasons, the drive for data sharing has been optimized by the increasing number of policy documents developed to facilitate this process (Besançon et al., 2021). However, data sharing must be governed according to ethical standards that ensure no risk of harm to participants and promote public trust in such endeavors (Staunton et al., 2019). In South Africa, this governance framework was recently established by the gazetting and enforcement of the POPIA (no. 4 of 2013). Questions have consequently been raised by researchers about the impact of POPIA on informed consent, standards of anonymization, secondary use of data, and privacy in open access.

A strict interpretation of section 13(1) of POPIA suggests that it is only permissible to request specific consent from participants. Under this interpretation, unless consent for sharing of specific data and/or samples is obtained at the onset of the study, it is not possible for researchers to engage in data/sample sharing practices (Staunton et al., 2019). Practically, this is considered inefficient and potentially wasteful. As such, POPIA permits the secondary use of personal information within the context of research without the need to obtain further consent if “the research is necessary to prevent or mitigate a serious and imminent threat to public health or safety” or “if the personal information will only be used for research AND it will not be published in an identifiable form”. As broad consent is a tool through which sample and data sharing has frequently been made possible, phrasing used within POPIA regarding informed consent has initiated a debate among researchers regarding the legality of broad consent (Staunton et al., 2019, 2020). The National Department of Health (DoH) Ethics in Health Research Guidelines defines broad consent as follows: “the donor permits use of the specimen for current research, for storage and possible future research purposes, even though the precise nature of future research may be unclear at present” (National Department of Health, 2022). In contrast to the strict interpretation of POPIA section 13(1), Thaldar and Townsend (2020) have posited that section 15 of POPIA makes provision for further research without obtaining new consent if the personal information collected previously or elsewhere is to be used for a specific purpose. Based on their interpretation, once specific consent has been obtained, researchers may continue to conduct their ongoing research under the provisions set forth in POPIA section 15 (Thaldar and Townsend, 2020). While there are enforceable conditions such as data security governance and participant risk of harm linked to this interpretation, from a practical perspective, this latter perspective essentially considers the receipt of specific consent as grounds for extended research privileges and some degree of broad consent.

Regardless of the argument made, until judicial case studies become available, the practical application of such clauses within POPIA remain debatable and open to interpretation. This is because POPIA-driven processing of personal information is principle-based, rather than sector-specific (Staunton et al., 2021). This has consequentially resulted in uncertainty regarding the appropriate application of POPIA in relation to health information for research purposes. In order to provide clarity in the healthcare/medical research sectors, the Academy of Science of South Africa (2022) and several of its stakeholders commissioned the development of a Code of Conduct (Staunton et al., 2020, 2021). This Code aims to compliment POPIA and to provide sector-specific guidance on its interpretation and application. In so-doing, the Code aims to clearly communicate the expectations placed upon researchers when working with health information or engaging in health-related research. The final draft of the Code of Conduct is currently being finalized (Academy of Science of South Africa, 2022).

### Research ethics

In addition to fulfilling POPIA requirements, scientists are equally bound by research ethics. This is important given that researchers are increasingly applying open science principles and making anonymized data available for analysis *via* publicly accessible repositories (Besançon et al., 2021). Data that is ethically the most sensitive can sometimes be the most valuable, and the ability to utilize it depends on the ability to preserve the privacy of the research subjects (Dennis et al., 2019). Research ethics committees (RECs) or similar regulatory bodies are tasked in the same way as their legislative peers with ensuring that no harm comes to research participants as a result of data sharing. Researchers are therefore not only bound by considerations of legislation, but also by the interpretation of the legislation by RECs. This may create a scenario where a REC may not approve research activity to satisfy open science principles, including open data, open source, and sample sharing, even though provisions are made for this under the legislative framework. It is therefore to the benefit of researchers that they are cognizant of this fact and work in collaboration with those able to provide legal and ethical guidance during the construction of their research protocols. This is particularly important in LMICs where resources needed to repeat certain aspects of their studies are often lacking due to legal or ethical constraints.

Researchers should similarly be sensitive to the fact that once data is shared, it is very hard to take it back. Additionally, despite the obvious ease of identifying research participants using personal information such as names and addresses, it is possible to reverse-engineer an identity from a wide variety of anonymized sources (Narayanan and Shmatikov, 2010). To protect sensitive data from unauthorized use, computational analysis must be accompanied by strict access control mechanisms and non-technical measures such as informed consent. It is considered unethical to upload data that has not been anonymized; recruiting research participants would not be permitted if this were not done (Dennis et al., 2019). As such, open sharing of sensitive data may be deemed illegal and may hold dire consequences for those who partake in such practices without adequate authorization (Dennis et al., 2019). Problems associated with data sharing are therefore critical to answer, especially for those engaged with qualitative research and associated data (Kirilova and Karcher, 2017).

Because sophisticated reverse-engineering identity techniques may allow for the re-identification of research participants, it is not possible to guarantee anonymity. While it has been suggested that data should never be shared, many are in favor of sharing and are actively engaged in developing policies and processes that would facilitate data sharing through ethically and legally sound means (Kirilova and Karcher, 2017). With regard to privacy, key issues such as the nature of the consent and who owns the data must be considered. This is important since most research institutions claim ownership of data collected by their researchers (Dennis et al., 2019). It is recognized however that research participants remain the owners of their information, even if collected for a research study, and therefore have the legal and ethical right to request that their data be amended and/or deleted without question or consequence. However, in practice, few participants execute their authority to do so largely due to logistical barriers. In addition, some researchers treat data like they own it and retain the data upon moving between institutions. In extreme and limited cases, while lacking the appropriate institutional approval to do so, researchers may publish the data on open platforms (Dennis et al., 2019). Some open data policies permit the secondary use of research data. Under these circumstances, data is used by researchers not involved in obtaining the initial consent for purposes and studies outside of the initial consent (Cummings et al., 2015). Research participants are not necessarily informed of this practice. Furthermore, research participants are not informed of the purpose for which their data will be reused under these open data policies. Such policies may have an impact on obtaining informed consent, especially if potential participants refuse to participate because of these open data policies, which may result in unreliable sample information and databases (Cummings et al., 2015). This may further result in legal consequences such as fines and criminal penalties, violations of ethical standards or data protection regulations that may result in irreparable damage to a provider's reputation (Wiesenauer et al., 2012).

It is thus imperative that the principles that govern ethical open science practices, including the sharing of samples and data, be observed for all data in order to experience the maximum benefit from such data and information while ensuring protection of the research participants (Martani et al., 2019). When it comes to secondary use of data, the risk-benefit considerations must be balanced so as to provide a useful resource to others while limiting the risk of exposure to participants. This is particularly important in health research as the secondary use of data increases the range of research projects that can be conducted, reduces not only the time spent on projects but also the operational and research costs, and increases the capacity of healthcare professionals to make evidence-based decisions for the continued improvement and delivery of good quality healthcare. Martani et al. (2019) have reported three categories of cutting-edge research initiatives within the healthcare sector. These include reusing data for: (1) “genomics and environmental health research;” (2) “clinical research in order to more rapidly identify and potentially recruit research participants;” and (3) “retrospective comparison of data from patients that have received conventional or alternative treatments, respectively.”

## Open science: Other barriers and misconceptions

The cost of accessing subscription journals, as previously discussed, is one of the most prominent barriers to dissemination of research findings in LMICs and may prevent research from being accessible to scientists and the public alike (Newton, 2020; Kwon, 2022). This challenge has however been exacerbated by the shift to online and open access publication models, given the difficulties that LMICs may experience with access to the Internet. When Internet access is possible, it still remains expensive and sometimes unstable within LMIC settings. Another barrier to open access for LMICs is the exorbitant and often prohibitively high APCs/publication fees which researchers or their associated institutions are unable or unwilling to pay. Many of these fees are more than the annual subscription to the journal, and often exceed the monthly salary of a researcher. Some researchers from LMICs are exempt from fees, but this is often reserved for the countries with the lowest gross domestic products and weakest economies. Many open access publishers impose a delay thereby decreasing the immediacy of the research or an embargo period, for example, 6 months for “green” open access. As indicated by Mwelwa et al. (2020), other barriers to open access and open science in Africa can broadly be summarized as deficiencies or limitations with regard to governmental or political, regulatory, institutional, financial, and researcher-centric structures. These categories include those challenges created by a lack of resources, such as access to research databases and journals, human capital and information and communication technology infrastructure, as well as the distrust or concerns that researchers may have regarding the ownership of published findings and any subsequent product developments. This latter point also concerns academic institutions and funding agencies, notably those located in Africa, who are adversely affected by the costs of APCs for reasons previously discussed. These challenges are perpetuated at government level owing to “a lack of political commitment in governments” and “a lack of national and institutional policies to provide a legal and regulatory framework for open science” (Mwelwa et al., 2020). In South Africa this problem is being addressed through a National Open Science Policy currently in draft form (Research Professional News, 2022).

### Perception of research quality

The initial perception was that open access publication and research would be of lower quality. As open access has expanded, misinformation and concerns have decreased and most researchers now have positive attitudes to open access publishing (Nobes and Harris, 2019). The quality of open access publishing would only be compromised if journals did not follow a rigorous peer-review process. Authors should choose reputable journals for open access publishing. Predatory and fraudulent journals do not provide the same quality publications as reputable journals and should therefore be avoided.

It is well-known that articles published in subscription-based journals are initially only visible to people at institutions which have a license for these specific journals and are thus less visible than those published in open access journals. Despite this well-known fact and as measured through citations, researchers do not necessarily prioritize publishing in open access journals (Perianes-Rodr-Guez, 2019).

To increase the visibility and credibility of research findings, researchers may make research data available in open access repositories (Misgar et al., 2020). The Registry of Research Data Repositories provides an overview of repositories available for research data across all academic disciplines and is funded through the German Research Foundation (Registry of Research Data Repositories, 2022). In 2019, the registry indexed 2264 repositories with a metadata description (Misgar et al., 2020). Digital Object Identifiers (DOIs) are used to access and cite registry records. Open access research data repositories have also been developed by Brazil, Russian Federation, India, China, and South Africa, an association of five countries also referred to as the BRICS (Misgar et al., 2020). The highest number of repositories is found in China (81), followed by India (51), Russian Federation (23), Brazil (18), and South Africa (15). English is the common language among all BRICS countries.

## Getting practical: Real-world impact of APCs

The previous sections have provided background on open science, and in particular open access. The COVID-19 pandemic has revealed that open access is inequitable, especially as far as LMICs and UMICs are concerned. The opportunities of preprints and challenges with predatory journals and research quality are briefly discussed. However, the core of this paper exposes the challenges that authors/researchers experience with high APCs and the resultant profits that publishers make. The role of higher education and research institutions in providing resources for authors/researchers are explained with the emphasis on UMICs, especially South Africa. Privacy and research ethics in medical research becomes challenging in open data sharing environments and need to be strictly regulated. The following is an overview of how researchers at the ICMM at the University of Pretoria manages the high APCs and their choice of journals with limited research funds.

**Figure 1:**
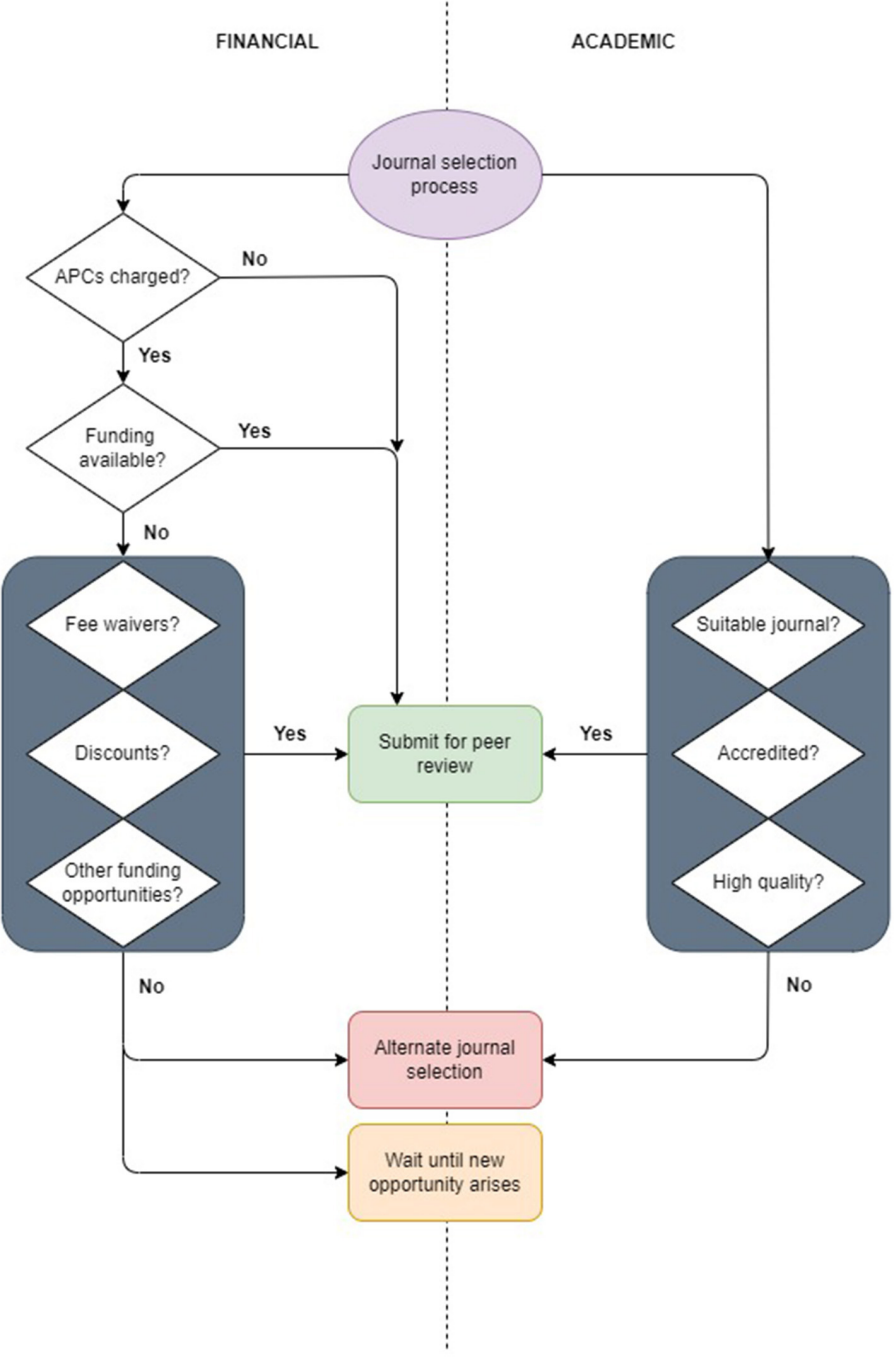
Journal selection algorithm. The arrows indicate the journal selection process when APCs are charged vs. when no APCs are charged from a financial and academic perspective. APCs, article processing charges. Image created by JVR/JM using draw IO network diagrams (https://app.diagrams.net/).

From a financial perspective, the first question asked concerning a manuscript is whether or not there are APCs. Briefly, if no APCs are charged, assuming that the journal meets the various quality standards set by the research industry and/or institute (Figure 1), authors will proceed to submit manuscripts for publication. If APCs are charged, several additional questions are asked prior to submission of the manuscript. These include whether there are fee waivers, fee discounts or other funding opportunities available for publications. Where a suitable journal cannot be selected owing to cost or lack of funding, lower APCs, or no APCs may be considered instead. Should no suitable journal be found for the manuscript, submission of the manuscript may be delayed until a financially suitable publication option becomes available.

**Figure 2:**
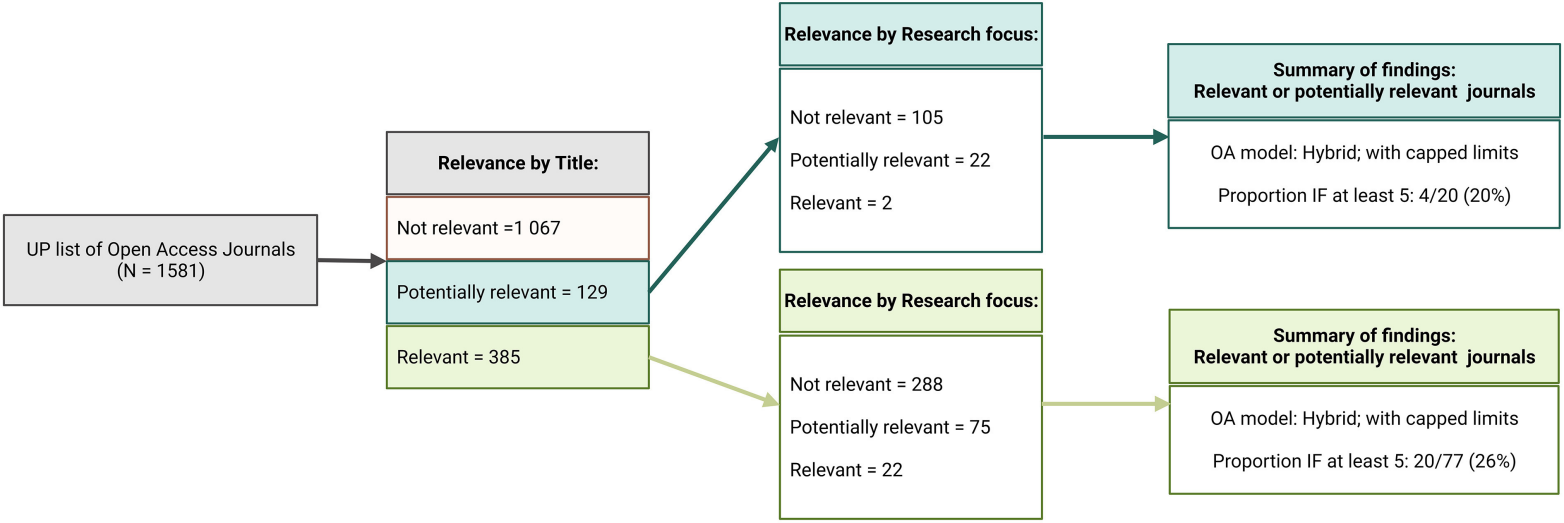
Open access journals with fee waivers considered to be relevant or potentially relevant to research conducted at the ICMM. UP, University of Pretoria; OA, open access; IF, impact factor. Image created by JVR/AA using BioRender (https://Biorender.com/).

From an academic perspective and as illustrated in Figure 1, manuscript submission is determined by journal suitability, accreditation and quality (as measured through the IF). While subject specificity is evaluated through journal titles and research focus areas (Figures 1, 2), at the University of Pretoria, journals are considered to be accredited if they appear on lists generated by Clarivate Analytics Web of Science, IBSS, the South African DHET, Norwegian, SciELO SA, Scopus, and the DOAJ. This typically results in a list of several potential journals that are further evaluated according to their IFs. Traditionally, journals with higher IFs are believed to publish research of higher quality and are therefore more likely to gain greater research exposure and readership. As reported by Alberts (2013), there are unfortunately evaluation structures that consider journals with IFs less than 5.0 to be “of zero value”. While this is largely dependent on the field of research and data used to determine such metrics, journals with IFs of at least 5.0 are meant to represent the top 10–20% of all journals within the medical sciences and may therefore be seen by some as an arbitrary benchmark of “good quality” (Alberts, 2013). Clarivate's Journal Citation Reports are often used for this purpose (Clarivate, 2022). While this standard is not strictly adhered to within the ICMM, journals with higher IFs will nevertheless be favored over lower IF journals for manuscript submission (Figure 1). Assuming that the academic and financial components have been adequately met, the manuscript may be submitted for review.

Practically applied, the University of Pretoria's Library Services website provides a list of 1,581 journal titles associated with fee waivers for open access (Figure 2). Considering that the ICMM conducts inter- and cross-disciplinary research, by observing the journal title alone, 129 (8.2%) and 385 (24.4%) journals would be perceived as being potentially relevant or relevant for manuscript submission, respectively. At the ICMM, journals focused on stem cell research, obesity, diabetes, cancer, cystic fibrosis, hypoxic ischemic encephalopathy, human immunodeficiency virus, human leukocyte antigen studies, and genetic susceptibility to disease would be considered from a cellular and molecular perspective. Where research has been conducted in cross-disciplinary fields, relevant law, computational biology, and engineering journals may also be considered. As such, when considering these potential journal titles based on research content, only 121 journals (7.7%) would be further considered for publication. Of these, impact factors were only available for 97 journal titles (6.1%), with a total of 24 journals (1.5%) having an IF of at least 5.0 (Figure 2, Table 2). While the median and mean IFs were respectively found to be 3.617 and 4.409, the maximum and minimum IFs were noted as 25.113 and 0.910, respectively. Each of these titles are associated with a hybrid open access model with “first come, first served” capped limits to fee waivers.

**Table 2:** The trends in impact factor relative to journals marked as potentially relevant or relevant to researchers based at the ICMM.

**Impact factor details**	**Potentially relevant journals**	**Relevant journal**	**All journals**
N (IF <5.0)	60	13	73
N (IF ≥ 5.0)	19	5	24
N (IF not reflected)	18	6	24
Minimum	0.91	1.615	0.910
1st IQR	2.452	2.087	2.447
Median	3.780	3.587	3.617
Mean	4.419	4.365	4.409
3^rd^ IQR	4.972	4.971	4.982
Maximum	25.113	11.598	25.113

IQR, Interquartile range; IF, impact factor.

While the finer breakdown of this exercise can be seen in both Figure 2 and Table 2, it must however be noted that this form of evaluation is subjective in nature, so there may be some differences between what one researcher notes as potentially being of value compared to another. This may similarly be extended to what is understood by “predatory journal.” In the context of this exercise, a predatory journal represents a journal that is (1) not accredited by the University of Pretoria standards previously indicated; (2) requests payment for manuscript review; (3) does not offer peer-review or offers sub-standard peer-review; (4) has a very low impact factor; and (5) promises rapid manuscript review, acceptance and publication. Regardless of these points, following the examination of the open access journals for which fee waivers were noted according to the list available through the University of Pretoria's Library Services website, it is important to note that capped limits for hybrid open access journals exist for all journals that would potentially or definitely be considered for manuscript submission by researchers at the ICMM. While it is not publicly known whether the capped limits are per faculty, per academic institution, or per country, or whether the limits are set to one, ten, 100 or 1,000 or more publications, that they are hybrid journals in nature *per se* is not of concern. What is of concern is that the University of Pretoria (and other academic institutions in South Africa) does not contribute to APCs for hybrid journals. Recently signed agreements between select publishers and research institutions like the University of Pretoria and others in South Africa have created an opportunity for complete fee waiving on select hybrid journals (UP news, 2022). This is of significant value to South African researchers operating within participating institutions (such as the University of Pretoria) and has the potential to have far-reaching benefits to individuals in public and private entities.

## Solutions to the challenges

Approximately a third of global research articles are now being published as open access and there is a strong drive to further increase this number (STM Global, 2021; Delta Think, 2022). Currently, the peer-review process has little benefit for the reviewer. Some journals offer incentives and rewards to reviewers such as subscription access for a limited period of time (The Conversation Africa, 2022b). This is not ideal for universities as it only benefits the individual reviewer. Instead, publishers may consider a voucher approach where vouchers are provided to reviewers' institutions. In LMICs this may contribute toward journal subscription costs or APCs and may also encourage academics to be involved in the review process. The high APCs associated with open access publishing remain a challenge for researchers in LMICs (Kwon, 2022). However, there are some options to consider when APCs are required and funding is limited; these include: (1) asking the publisher whether the journal would consider waiving or reducing APCs for researchers in LMICs; (2) enquiring whether the representative institution maintains an agreement with certain publishers that will allow publishing for free or at a discounted rate; and (3) enquiring from funders about the availability of funds for publications related to awarded grants. Publishing in societal journals is another potential solution as profits from these journals are re-invested into supporting a wide range of research activities. Researchers should also include publishing costs in grant applications. This is already encouraged by some funders such as South Africa's National Research Foundation. Other proposed solutions include increasing government subsidy for universities to aid in covering full APCs. Current government subsidies received by academic institutions to cover publication costs are divided amongst the Institution, Faculties, Schools and Departments, with a very limited amount of funding trickling down to researchers on an *ad hoc* basis. The full subsidy or a portion of this should in all fairness be returned to researchers to cover the costs of future publications. Additional agreements should be put in place between universities and publishing houses to assist researchers in LMICs to publish high quality research in reputable journals.

In relation to cost and in order to address some of the current pressures, Plan S and several other initiatives have been established to increase open access. Plan S is an initiative that was implemented by Science Europe, a group of state funded scientists and researchers from 12 national European funding agencies referred to as “cOAlition S” (Plan, 2022). In 2021, as part of Plan S, a group of international funders (including UK Research and Innovation, the Bill and Melinda Gates Foundation, the Wellcome Trust, and the World Health Organization) launched a major reform of the way funded research is published (Mering, 2020; Shamseer et al., 2021). Recipients of grants funded by agencies affiliated to Plan S are required to publish research in open access journals or platforms and make publications available *via* open access repositories (Plan, 2022). In order to facilitate this, funders will pay APCs (up to a limit still to be determined) to “gold” open access journals. Furthermore, Plan S supports the retention of publication copyrights by authors and their affiliated institutions, as well as the use of open licenses (Mering, 2020). Although the hybrid journal publishing model is not supported, transformative journal agreements are provided as an option to gradually increase the amount of journal open access content. The aim is for all journals to be open access by the end of 2024. Among the few non-European agencies, the South African Medical Research Council has also joined Plan S and may serve as an early indication of how Plan S will operate in LMICs (The Scientist, 2022). Some requirements of Plan S already encourage more equitable publishing practices. Open access journals or platforms publishing results generated using funds from Plan S signatories should provide APC relief either through waivers or discounts for researchers from LMICs (The Scientist, 2022). Some researchers have suggested that Plan S should support “diamond” open access journals, allowing free reading and free publishing. These journals are often supported by scholarly societies, receive funding from higher education institutions, and the editorial boards consist of volunteer editors (The Scientist, 2022). According to Robert Kiley, the head of strategy of cOAlition S and the head of open research development at the Wellcome Trust, publishers are required to share their pricing and service data with cOAlition S, starting in July 2022. Incentives will be provided for publishers to do so, such as continued funding by cOAlition S to cover open access publication costs. In the future, researchers should be able to choose to pay only for essential publication services and should be exempt from covering marketing and other non-essential costs (The Scientist, 2022). There are various other open access projects under the SHERPA (Securing a Hybrid Environment for Research Preservation and Access) organization (SHERPA, 2022), UNESCO's Open Science project (UNESCO, 2022b) and Research4Life (R4L) (Research4Life, 2022), formerly the WHO Hinari Program.

According to the journal Science, authors of research papers will be allowed to share an almost final version of their articles in a public repository of their choice without paying any fees from January 2023 (Else, 2022). The policy will apply to all five subscription journals in the Science family. Currently, most authors can share their accepted articles only in an institutional repository or on a personal website. Authors have an embargo period of 6 months after publication before they can add their papers to other repositories, such as PubMed. However, there are exceptions for some authors supported by funders from cOAlition S.

## Concluding remarks

Limited access by researchers in LMICs and UMICs to the latest research information affects their ability to provide quality work of current relevance, and this barrier must be lifted. Open access serves to bridge this gap and will provide equity in the research space. The National Research Foundation's mandate is to contribute to national development by:

- “Supporting, promoting and advancing research and human capacity development, through funding and the provision of the necessary research infrastructure, in order to facilitate the creation of knowledge, innovation and development in all fields of science and technology, including humanities, social sciences and indigenous knowledge”;
- “Developing, supporting and maintaining national research facilities”;
- “Supporting and promoting public awareness of, and engagement with, science; and”
- “Promoting the development and maintenance of the national science system and support of Government priorities”.

In the present technology driven era, it is critical to strengthen open access initiatives and to transform journals, platforms and repositories to make research freely available online. This will enable sharing of knowledge and enhance global communication, while improving research potential and visibility of institutions and researchers. The increase in predatory journals and misconceptions regarding open access are challenges that need to be overcome in order for open access to achieve its full potential. In order for this endeavor to be successful in LMICs and UMICs (such as South Africa), governments and funding agencies need to adopt and improve open access initiatives in support of African research. The number of open access journals that offer fee waivers for LMICs should be increased to broaden journal options for publication. Until all countries and scientific communities have equal access to all research available, the impact of open access will remain limited and inequitable.

## Author contributions

AS and MSP completed the final preparation and editing of the manuscript. JVR, JM, and AA created the tables and figures. All authors contributed to the article and approved the submitted version.

## Funding

This work was supported by the South African Medical Research Council Extramural Unit for Stem Cell Research and Therapy and the University of Pretoria through the Institute for Cellular and Molecular Medicine. AS, JM, JVR, and MSP receive funding from the Bill and Melinda Gates Foundation (INV-022216).

## Conflict of interest

The authors declare that the research was conducted in the absence of any commercial or financial relationships that could be construed as a potential conflict of interest.

## Publisher's note

All claims expressed in this article are solely those of the authors and do not necessarily represent those of their affiliated organizations, or those of the publisher, the editors and the reviewers. Any product that may be evaluated in this article, or claim that may be made by its manufacturer, is not guaranteed or endorsed by the publisher.
